# Vulnerability assessment of debris flow in the central Peruvian rainforest – An intercultural approach

**DOI:** 10.1016/j.heliyon.2023.e20788

**Published:** 2023-10-11

**Authors:** Luis Izquierdo-Horna, Angelica Sánchez-Castro, Jose Duran

**Affiliations:** Department of Engineering, Universidad Tecnológica del Perú, Lima, Peru

**Keywords:** Disaster risk reduction, Debris flow, Interculturality, Social vulnerability, Peru

## Abstract

For several decades, calamitous debris flows have inflicted profound negative impact on Peruvian rainforest society, encompassing both economic losses and human casualties. To address this concern, this study proposes a methodological tool to assess vulnerability while focusing on intercultural nuances. The contextual analysis of the incident reports identifies La Merced (Junín, Perú) as a severely affected locality, thereby justifying its selection for a detailed case study on the Pampa del Carmen sector. The study conducted a thorough systematic review of parameters such as diglossia, poverty, and origin that are crucial for vulnerability assessment. Moreover, these parameters aided in developing a structured digital survey. The integration of survey data into the analytic hierarchy process revealed high levels of vulnerability in the sector, emphasizing the imminent need for targeted interventions. The intercultural approach is significant as it facilitates future risk mitigation strategies based on effective integration and genuine acknowledgment of social dynamics and individual freedoms within the region for devising impactful risk management policies and plans.

## 1 Introduction

Owing to the interplay of physical and geospatial conditions along with anthropogenic activities [1], Peru remains vulnerable to a range of socioecological disasters, which exert substantial impacts on populations, infrastructure, and economic endeavors [2]. In the past five years, Peru has recorded approximately 35,000 emergency situations, a significant portion of which can be ascribed to hydrometeorological events and external geodynamic occurrences [3]. Based on this scenario, a considerable number of these emergencies have been attributed to landslides, especially debris flows. In case of landslides, a debris flow represents the swift, potent, gravity-induced downhill movement of water-soaked debris composed of soil, rocks, and vegetation. This phenomenon transpires when copious rain saturates unconsolidated materials, forming a perilous mixture that can cause erosion, damage to property and infrastructure, and endanger human lives [4]. These events, originating from the intricate interplay between natural forces, contribute significantly to the challenges encountered by various regions within the Peruvian territory. Furthermore, these challenges can be influenced by external factors (e.g., climate, temperature) [5] or other natural phenomena (e.g., intense precipitation and earthquakes) [6] that may potentially escalate the risk and vulnerability levels [7]. Thus, the vulnerability levels of certain sectors must be determined to safeguard the livelihoods and living conditions of citizens.

Vulnerability studies are indispensable for enhancing the adaptive and responsive capacities of populations exposed to various natural events across diverse contexts [8]. To this end, Guillard et al. [9] advocate for assessing vulnerability by addressing the disparities among susceptible groups, incorporating factors such as ethnicity, education, wealth, and healthcare access in a unified vulnerability map. As such, adopting an intercultural perspective is beneficial as it aligns philosophical notions of well-being with the identification of deprivations experienced by populations [10]. Moreover, Cutter [11] underscores the increasing importance of social vulnerability assessments, including age, status, gender, and household composition as pivotal indicators. In addition, these assessments embrace concepts such as resilience, mortality, and occupation [12]. Thus, understanding the vulnerability within multicultural and pluricultural populations is essential, and intriguingly, poverty emerges as a recurring socioeconomic factor governing vulnerability [13]. Here, poverty is not only constrained to economic deprivation but also signifies a deficit in capacity development [14]. This study proposes a cross-sectional evaluation methodology centered on interculturality to assess vulnerability through an analytical hierarchy process (AHP) aimed at building resilient communities for contributing to risk prevention and mitigation from an engineering perspective, specifically in the context of debris flow.

Given these complex interconnections, understanding the concept and dimensions of vulnerability, with emphasis on social vulnerability, becomes crucial. For instance, social vulnerability frequently equates to poverty [13], representing the difficulties encountered by communities due to adverse socioeconomic conditions and limited resources post disasters and calamities [15]. In addition, social vulnerability often emerges due to the deterioration of family and community institutions, as well as insufficient government protection and security measures [16]. To completely contextualize the social vulnerability experienced within a specific segment of society, the developmental paths of individuals within their surroundings should be considered [17]. This involves investigating potential underlying causes, including the scarcity of economic resources, restricted access to political power and representation, and evolving cultural norms and practices. Thus, these factors can evolve temporally and differ across various spatial dimensions. Additionally, anthropogenic factors such as age, race, ethnicity, and gender can considerably influence these causes [18]. This perspective aligns with that of Cutter et al. [19], who reported that vulnerability holds diverse connotations based on the orientation and viewpoint of the research. Therefore, these assessments can identify the conditions that render individuals or places vulnerable to extreme situations.

For quantification, Pérez et al. [8] asserted that social vulnerability can be appraised using both qualitative and quantitative perspectives. The former encapsulates subjective factors that are often elusive to objective quantification, whereas the latter entails measurable, extrapolable, and verifiable outcomes. Qualitative data collection involves personal interviews and expert insights [20,21], whereas quantitative approaches rely on probabilistic sampling and observational records, exemplified by methodologies such as the hazards of place model (HPM) developed by Cutter [22]. Furthermore, Cutter et al. [23] stated that biophysical and social vulnerabilities interact dynamically with each other and collectively influence risk mitigation. In general, the HPM method has been extensively applied to assess social vulnerability against natural phenomena [[24], [25], [26]]. Moreover, Perles [27] suggests that social vulnerability can be described using variables related to exposure and possible losses through georeferencing [28] or statistical techniques [29]. In this context, principal component analysis technique is notable for its contribution to positive data processing outcomes by converting qualitative variables into quantifiable entities [30]. This transformation establishes the foundation for constructing indicators with balanced weighting [31]. Similarly, an alternative approach involves the use of artificial neural networks (ANN), which impose certain limitations such as the limited predictive capabilities and the requirement for substantial datasets [32]. Furthermore, the development and implementation of ANN necessitate an investment of substantial time owing to the extensive prerequisites regarding training data [33].

Nonetheless, addressing the persistent challenges of acquiring accurate information remains a recurring theme across all aforementioned methods. Consequently, assessing social vulnerability in the context of debris flows might become complicated owing to a variety of technical and logistical constraints such as regional linguistic limitations [34], hard-to-reach settlements [13], or the absence of community training provided by municipalities [35]. In addition, the information is not typically acquired using an inclusive approach that encompasses individuals from diverse environments. Thus, an intercultural dimension must be imperatively identified within the amassed data [36,37], which promotes an equitable distribution of coexistence responsibilities among groups inhabiting the same space and to safeguard their economic, cultural, and linguistic rights [38]. In this context, interculturality can be defined as the interaction between cultures, accentuating mutual exchange and coexistence within a framework of respect and tolerance [39,40].

Finally, this study aims to develop a vulnerability index focusing on intercultural considerations in the sector. This comprehensive approach entails an AHP, which validates the weighting of vulnerability parameters according to the subjectivity of judgment [41]. Upon accommodating regional spatiotemporal disparities, this methodology can be adapted to various territorial scales, and in this study, it was validated with a case study on a historically significant community in the central Peruvian rainforest (Pampa del Carmen, La Merced, Junín). Overall, this study aims to support the formulation of public policies and augment the comprehension of population dynamics over time.

## 2 Central Peruvian jungle - Main features

The central Peruvian jungle is located in the central area of Peru, encompassing the provinces of Satipo, Chanchamayo, and Oxapampa [42], with a total population of 507,051 individuals [43]. The soil is primarily composed of clay and sand that tend to detach quickly, thereby causing landslides and debris flows [44].

### 2.1 Intercultural features

Chanchamayo is a city situated within the Junín region, known for its long-standing history of hosting both foreign citizens and individuals from other regions of Peru. According to the 2017 census, 83.7 % of the surveyed population were native residents of the Junín region, 16.2 % migrated to Junín, and 0.1 % were migrants from another country [3]. Linguistically, Spanish is the predominant language, followed by five additional languages: Ashaninka, Kakinte, Nomatsigenga, Yanesha, and Quechua [45]. According to the National Institute of Statistics and Informatics (INEI for its Spanish acronym) [46], 29.96 % of the Junín population pertain to Quechua, 0.38 % are Aymara, 6.21 % are Amazon natives, 0.06 % are indigenous, 1.90 % are Afro-Peruvian, and more than 50 % is primarily mestizo. In terms of cultural diversity, the study population manifests a diverse array of beliefs, values, principles, and worldviews.

### 2.2 Social features

According to the 2017 census, the district of Chanchamayo reports a total of 27,790 citizens among which 83.18 % reside in urban regions and 16.82 % in rural areas [46]. The urban dwellers predominantly inhabit stand-alone houses, manors, tenements, settlements, and shanty towns, whereas the rural population resides in shacks, cottages, and small self-constructed houses [46]. Regarding public health, the local population predominantly suffers from respiratory and parasitic infectious diseases, mainly affecting children and the elderly, which can be attributed to the limited accessibility to essential services such as drinking water, energy, and drainage [47]. In contrast, according to the 2017 census, only 46 % of the Junín population completed their secondary education, 18 % completed primary education, which are below-average percentages. Furthermore, only 12 % of the population completed higher or university education, and only 1 % completed their graduation program, which are strongly discouraging academic statistics [3].

### 2.3 Economic features

Approximately 70 % of Chanchamayo's population is engaged in farming papaya, oranges, coffee, and passion fruits, among other crops, and the remaining 30 % are occupied in garment sales, handicrafts, restaurants, etc. [35]. In addition, the per capita income of the population ranges from $185–350 [48]. Thus, the agriculture sector employs the largest portion of the economically active population, whereas and its smallest portion is constituted by technical professionals [46]. Furthermore, poverty statistics from 2009 indicated that 12.4 % of the total population lived in poverty, with 1.7 % living in extreme poverty and the remaining 10.7 % living in non-extreme poverty. However, the per capita income of 16.1 % of the population is insufficient to meet basic needs such as food, health care, transportation, and education [46].

### 2.4 Environmental features

The district of Chanchamayo experiences a cool, mesothermal climate with an average annual temperature of 30 °C, which can decline to 18 °C during the monsoon from November to April. During this period, the precipitation ranges from 1200 mm to 2400 mm, with an average annual precipitation of 1170 mm/year [49]. In terms of soil composition, the district features a conglomerate arrangement typical of a piedmont setting, comprising a variety of rock types including limestones, granites, sandstones, and metamorphic rocks. These rocks are characterized by subrounded to rounded shapes and can have diameters up to 50 cm. Moreover, they are enveloped within a matrix of silty–sandy material, which is bound either by clayey or calcareous cement [35]. Furthermore, Chanchamayo hosts cliffs and mountains covered in sedimentary rocks and steep slopes (i.e., >45°) [44]. Until 2018, approximately 43,967 ha of humid forests have been deforested, thereby exposing a large portion of the territory to landslides [50].

### 2.5 Political aspects associated to disaster risk management

According to the findings from the Geological, Mining and Metallurgical Institute (INGEMMET for its Spanish acronym) [35], the municipality of Chanchamayo organizes geological field inspections in collaboration with local residents and authorities to effectively mitigate, prevent, and minimize risks associated with geological and natural hazards. Accordingly, a systematic monitoring regime has been implemented for areas designated as critical. This proactive approach is inspired by the identification of potential natural hazards and disasters, aiming to refine and extend mitigation strategies [51]. Nevertheless, these efforts have yet not been able to achieve significant progress, considering that local authorities have encountered challenges to foster a culture of preventive practices [52]. This deficiency is evident in the limited awareness among the community toward the potential risks. Notably, only 43 % of the population demonstrates the capability to effectively respond during emergencies. To address this shortfall, various legislative measures have been enacted with the dual purpose of increasing public awareness and ensuring the standardized execution of disaster prevention drills [53].

### 2.6 Physical characteristics of local housing

According to the data provided from INEI [46], the district of Chanchamayo presents a varied housing landscape in which a substantial number (78.6 %) of dwellings is constructed using durable materials such as clay bricks or cement blocks. Interestingly, a smaller proportion (1.9 %) adheres to traditional methods, utilizing adobe or rammed earth walls, whereas an additional fraction (4.5 %) utilizes less sturdy materials such as matting or cardboard. This diversity in construction materials reflects a confluence of modern and traditional influences, contributing to the distinctive architectural fabric within the district. Furthermore, certain inadequately constructed residences are situated on challenging-to-access elevated terrains owing to their steep inclines (17°–45°) and distinctive topographical attributes.

## 3 Case study background: Pampa del Carmen sector

This research focuses on assessing the vulnerability of the Pampa del Carmen sector, situated within the Chanchamayo district and province, to hazards of debris flows. The aim is to appraise the vulnerability of the community and devise strategies for mitigating the impact of such events in the future. Pampa del Carmen, located at an elevation of 775 m above sea level, experiences consistent heavy rainfall throughout the year owing to its wetland characteristics [46]. This situation is exacerbated by the presence of steep hills susceptible to river-induced slope destabilization, rendering the area as a breeding ground for debris flows [51]. Notably, Pampa del Carmen is bordered by numerous creeks and cliffs such as María Pía, Potoque, and Abanico [44]. Given the historical context and the geographical setup, this study specifically focuses on the María Pía creek, which meanders its course to the city of La Merced.

**Fig. 1:**
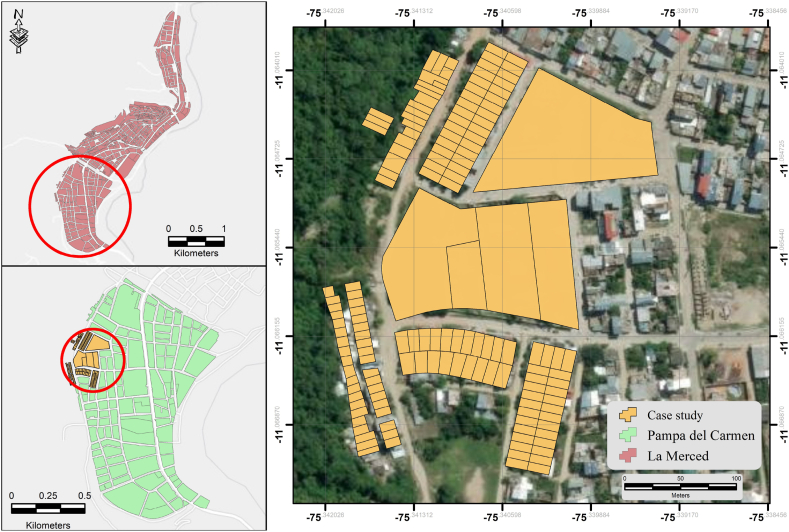
Geographical map of case study area—Pampa del Carmen Sector, Chanchamayo.

Historical evidence highlights a strong correlation between these debris flows and rainfall patterns. This combination, amplified by inferior soil quality and limited load-bearing capacity, contributes to substantial land shifts, thereby elevating the risk profile of the region. The adverse effects manifest in the destruction of residences, roads, and the safety of vulnerable populations, including individuals with disabilities, the elderly, the infirm, and children [54]. A recent incident on February 21, 2021 impacted houses and displaced construction materials along the 2-m-wide and 18-m-long course of the creek, which triggered an abrupt inundation of streets and surrounding homes [44]. Refer to Fig. 1 for a visual representation of the location of this present case study.

## 4 Materials and methods

This study employed an AHP framework, utilizing a comprehensive set of parameters to obtain a multifaceted representation of the study area. These parameters cover social, environmental, economic, and intercultural dimensions, collectively contributing to a holistic understanding of the vulnerability levels experienced by the local community toward natural phenomena. In principle, this research introduces a customized evaluation tool that is specifically designed for this geographical region. This evaluative instrument considers tangible parameters such as economic income and educational qualification, while incorporating intercultural facets like cultural diglossia, migration patterns, and the perception of the community toward its environment. Ultimately, the objective is to furnish governmental authorities with a robust foundation for devising mitigation strategies that can curtail the impact and losses sustained by the community. These strategies are designed to foster an inclusive environment while considering the diverse needs and perspectives of the population. Note that this research has obtained the necessary approval from the Research Ethics Committee of the Universidad Tecnológica del Perú (P-2021-LIM-27), confirming its commitment to ethical research standards. Additionally, all participants involved in the study have provided informed consent for using their information, affirming the ethical handling of data throughout the research process.

### 4.1 Data gathering

Upon examining the area of influence (Fig. 1) and utilizing a combination of local historical records and testimonies of residents, we contextualized and described the study area covering 138 dwellings pertaining to the Pampa del Carmen sector. Subsequently, by leveraging the information collected during the field survey, we defined the distinctive variables that characterized the case study. Intrinsic to this research, these descriptors were obtained by combining the authors' judgment with field observations. To address the methodological approach for acquiring data from the interview discussion, we employed both participant observation and interactive engagement. The data obtained from these dialogs were organized through a process of thematic analysis. Detailed notes and transcripts were compiled to capture the recurring themes, major concerns, opinions, and perspectives expressed in the interviews. Thereafter, these data were systematically reviewed to identify patterns and common issues. Based on this analysis, the potential indicators were extracted and selected based on factors such as their frequency, relevance to vulnerability assessment, and congruity with the scope of the research. Furthermore, a structured survey consisting a comprehensive set of 27 questions was designed to effectively collect pertinent information related a wide variety of aspects such as housing, access to essential services, income, health, age, language and education. All survey questions are listed in Appendix 1A in the supplementary section. Note that the head of household was the designated respondent for the survey. This inclusive approach contributed to a successful collection of 138 observations.

### 4.2 Analytic hierarchy process

The AHP method proposed by Saaty was selected to process the acquired information [55]. This technique facilitates the incorporation of all stakeholders into the decision-making process, enabling the measurement of criteria, evaluation and analysis of the problem by means of mixed comparisons (e.g., numerical or verbal). Additionally, the most representative advantage of this method pertains to the incorporation of qualitative aspects, which are often avoided due to complexity involved in their measurement. This facilitates a more comprehensive approach to the problem in an efficient and orderly manner. In addition, it aids in associating a specific topic by estimating its relative value through parameters to determine its significance (weight) [2]. In contrast, the AHP is based on pair-wise comparisons where each component reflects its weight with respect to additional problem items [56]. These pair-wise comparisons use the Saaty scale. The final product from this assessment yields the priority vector of the analyzed parameters [56]. Based on the prioritization vectors calculated for each variable, the weighted weights of each dimension were determined and the vulnerability levels were evaluated using the following expressions:

1 Vulnerability Level = F (physical dimension, social dimension, economic dimension)

2 Physical Dimension = F (physical exposure, physical fragility, physical resilience)

3 Social Dimension = F (social exposure, social fragility, social resilience)

4 Economic Dimension = F (economic exposure, economic fragility, economic resilience)

The vulnerability assessment equation (EQ1) applied to the selected sector encompasses a wide array of components, each of which is fundamental for comprehending overall vulnerability. The equation considers various elements that collectively contribute to vulnerability, including aspects related to the physical, economic, and social context of the case study, described in terms of exposure, resilience, and fragility. These specific components are included based on their direct relevance to the process of vulnerability assessment and their alignment with the unique characteristics of the case study area. These factors collectively contribute to the vulnerability landscape, providing insights into the intricate interplay between the environment, society, and the economy. The interdependence of these components highlights their significance in accurately capturing the multifaceted nature of vulnerability. Although the vulnerability assessment equation is inspired from established methodologies [2], it was employed to adopt the nuanced context and specific research objectives of this study. This adaptation ensured that the equations precisely encapsulated the dynamics at play within the selected sector.

## 5 Results

Through meticulous field observations and in-depth literature review [22,57,58], we identified a comprehensive set of variables to examine the case study. The detailed array of parameters and descriptors employed in this assessment—categorized under social, economic, and physical conditions—are summarized in Table 1. These dimensions were further assessed from the perspectives of exposure, fragility, and resilience. The AHP was implemented on this comprehensive dataset to ascertain and categorize the levels of vulnerability posed by debris flows within the study region. This process enabled the precise determination and categorization of the vulnerability levels associated with the study region when encountering a threat of debris flows.

**Table 1:** Variables used to determine vulnerability levels to debris flows in Pampa del Carmen Sector.

Dimension	Vulnerability factor	Variable
Physical	Exposition	Distance between the building and the risk zone
	Fragility	Building's structural system
		Structure's age
		State of conservation
		Building configuration
	Resilience	Use of urban equipment
Economic	Exposition	Electricity
		Water service
		Sewage service
	Fragility	Primary occupation of the head of household
	Resilience	Savings and insurance
		Access to household income
		Property rights
Social	Exposition	Population location
		Education level
		Access to health insurance
	Fragility	Age
		Disability
		Mother tongue
		Language used for everyday communication
		Place where has lived the longest
		Neighbor behavior
	Resilience	Training on disaster risk management

Furthermore, the insights obtained from the interviews and on-site observations revealed significant patterns within the population. Predominantly, the results revealed that the majority of inhabitants reside in single-story dwellings, often self-constructed around 30 years ago. A discernible trend of rectifiable wear and tear was observed, primarily attributable to the proximity of the María Pía creek. Alarmingly, there is a widespread lack of medical insurance coverage among the respondents, and their prevalent monthly income was below $300. The accessibility to fundamental government-provided services is remarkably limited. In terms of personal attributes, respondents commonly reported the absence of permanent disabilities and displayed a moderate level of academic attainment, with the majority completing only high school-level education. Age-wise, the surveyed individuals were primarily distributed across the age groups of 13–18 and 50–59 years. In terms of diglossia, Spanish is the common language along with Quechua and other pidgin dialects spoken throughout the area. Notably, these languages do not share phonetic or grammatical similarities. Furthermore, owing to the diverse origins of the settlements, they exhibited remarkable cultural diversity. Based on the collated information, the study identified three distinctive levels of vulnerability within the case study: medium, high, and very high. Detailed descriptions corresponding to each vulnerability level are exemplified in Table 2.

**Table 2:** Vulnerability levels stratification in the Pampa del Carmen area.

Vulnerability Level	Description	Range
**VERY HIGH**	The house is located in close proximity to the debris flow channel, i.e., within 300 m. Constructed using wood on land occupied over four decades, its decay tends it toward potential collapse. Rising to about five story, it offers only potable water service. The inhabitants, mostly very low-income (per capita income < $100) immigrants, are uneducated and use non-local languages. Age groups range from 0 to 3 years and over 65 years. A significant knowledge gap in disaster risk management is noticeable.	0.268 ≤ SV ≤ 0.485
**HIGH**	The dwelling is situated in proximity to debris flow events, ranging from 300 to 600 m. An unsteady brick house erected 30–40 years ago, its susceptibility to instability is heightened by the absence of preventive maintenance. Rising to about four stories, these buildings offer only drinking water and sewage services. The populace, predominantly low-income (per capita income < $150) immigrants, encounters educational deficits and communicates primarily in local languages. Age groups range from 4 to 12 years and 60–64 years. Minimal familiarity with disaster risk management is observable.	0.142 ≤ SV < 0.268
**MEDIUM**	The residence is situated at an average distance from debris flow incidents, ranging from 600 to 900 m. The brick-built house was constructed 20–30 years ago, with intermittent maintenance efforts at best. It reaches a height of roughly three stories, with accessible drinking water, sewage, and public health services. Generally, residents possess a moderate income (<$250); households comprise 2–3 individuals with higher education backgrounds, and most hail from nearby regions. Age groups encompass 13–18 years old and 50–59 years old. Communication primarily transpires in languages commonly spoken in the vicinity. Finally, basic knowledge of disaster risk management is evident.	0.068 ≤ SV < 0.142
**LOW**	The dwelling is located at a considerable distance from debris flow incidents (>900 m). It is a reinforced concrete house constructed within the past two decades. Regular preventive maintenance ensures its appropriate condition. With an approximate height of two stories, the structure is exclusively designated for residential use. The household benefits from comprehensive basic and educational services. Inhabitants earn a medium–high income (>$300), and households typically comprise 2–3 individuals. The age demographic ranges from 19 to 49 years. Communication primarily occurs in Spanish. Lastly, the residents bear a sufficient understanding of disaster risk management.	0.038 ≤ SV < 0.068

As listed in Table 2, the description of each vulnerability level was derived from an analysis of multiple factors relevant to the vulnerability assessment in the Pampa del Carmen sector. These factors included physical characteristics of the houses, their proximity to debris flow events, housing materials, maintenance, available services, socioeconomic conditions, population demographics, communication languages, and levels of disaster risk management knowledge. The description of each vulnerability level is a result of synthesizing various aspects related to the physical, socioeconomic, and demographic characteristics of the houses and residents. The age group information, level educational qualification, and per capita income serve as illustrative descriptors of the predominant groups within each vulnerability category, rather than implying exclusivity. The map categorizing the vulnerability levels in terms of dwelling incorporates multiple parameters to provide an accurate representation of vulnerability across the study area.

**Fig. 2:**
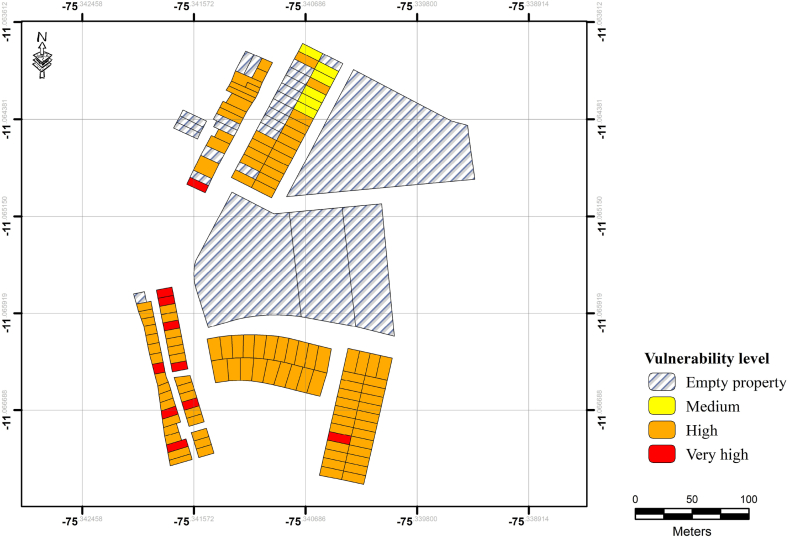
Case study vulnerability level – Pampa del Carmen Sector, Chanchamayo.

The georeferenced vulnerability levels derived from our case study are depicted in Fig. 2. In general, the area exhibits a predominant high level of vulnerability. Specifically, the buildings situated in proximity to the mouth of the María Pía creek, where the highest vulnerability levels are evident. Similarly, a substantial portion of the studied region demonstrates high vulnerability owing to the anthropogenic factors impacting the resident population. Correspondingly, the northwestern and southwestern sectors occupy higher-altitude and steeper terrains compared to the central and eastern areas. Nonetheless, the heightened vulnerability was not exclusively associated with higher elevations, but instead, was dependent on the prevailing conditions of both the housing infrastructure and households. Note that the validation of these findings was conducted in the field, yielding consistent outcomes. Note that, owing to the characteristics of the given sector, buildings displaying low vulnerability were not identified in the case study.

## 6 Discussion

Based on the comprehensive analysis discussed earlier, this section delves into a nuanced discussion of the key findings and their implications. For the decision-making process, the conducted AHP yielded results that align with the current conditions in the study area as it interrelated numerical and categorical variables to form unique data without excluding information. These results effectively represent the existing circumstances within our case study area, as corroborated by field observations that reinforce the absence of low vulnerability owing to the high exposure and fragility evident in both the houses under examination and the interviewed residents. In particular, the recognition and dissemination of the intercultural parameters such as diglossia and ethnicity presume a critical role in formulating public policies and prevention strategies. These parameters appropriately segment the exposed population, increasing the intersectional inequalities that contribute to heightened social vulnerability. Construction-related attributes are also considered in this dynamic. Upon implementing priority-ordered preventive and mitigative measures, the vulnerability levels are anticipated to decrease and reflect the current state of the population. This approach facilitates a deeper understanding of the interplay between social progress, individual autonomy, and communal well-being.

In contrast, upon evaluating the intercultural dimension, variables such as native language and place of origin underscore the significance of comprehending customs and traditions that impact disaster mitigation efforts. Notably, residences with dialects divergent from the hegemonic norm or originating from various regions exhibit elevated vulnerability levels. In addition, prior research demonstrated that Hispanic, Latino, indigenous or multiracial communities are typically exposed to higher vulnerability and limited adaptive capacity [13,25,58]. In this context, embracing an intercultural approach is essential for comprehending the multifaceted nature of the core issue delved into by this case study [36]. The intercultural dimension is pivotal for fostering a preventive and informed society, which is crucial for confronting diverse natural disasters [7]. Despite the awareness among residents, they persist in high-risk areas owing to economic constraints or familial commitments. The persisting significance of hazard comprehension, even in case of residents' awareness and their choice to inhabit high-risk zones owing to external factors, highlights a complex scenario. Although they acknowledge the risks, external limitations such as economics and family influence supersede immediate risk perception. This intricate interplay emphasizes that robust hazard understanding can be dominated by external pressures, which hinders effective risk mitigation strategies.

In terms of legislation, Peru operates within the Sendai Framework for Disaster Risk Reduction 2015–2030, prioritizing the prevention of new risks and the reduction of existing ones. However, this governance system needs to be further strengthened. Resilience management is essential for raising awareness among the population, as reported by Isnaini et al. [59]. Additionally, vulnerability serves as a cornerstone for risk reduction and territorial planning, reflecting the varying impacts of natural phenomena on multiple segments of the population. This highlights the persistent inequalities that render certain groups more vulnerable than others. The implications of the present findings are significant in a political context, elucidating the intercultural vulnerability originating from historical social processes of urban growth and accentuated by cultural diglossia. Therefore, the current results offer crucial political implications that should not be neglected, as the historical social processes of urban growth reflect a high degree of associated intercultural vulnerability accentuated by the existing cultural diglossia in the area. The resulting maps provide the empirical basis for promoting and guiding actions to understand and mitigate the existing inequalities.

## 7 Conclusions

This study aims to evaluate the levels of vulnerability in the Pampa del Carmen sector, with emphasis on the intercultural aspect, given the diverse cultural backgrounds and social dynamics in the area. To this end, each dimension of vulnerability—physical, socioeconomic, and sociocultural—was examined via exposure, fragility, and resilience. The selected descriptors derived from the fieldwork revealed that a significant portion of the analysis area is marked by high vulnerability to debris flow. The interplay of historical social processes of urban expansion further accentuates the intercultural vulnerability, especially evident in the outskirts of the city where cultural diglossia prevails.

Although the Pampa del Carmen sector has encountered various incidents of debris flow, those within the past five years are concerning because of their significant economic impact. Nevertheless, an optimal level of preparedness for forthcoming events remains elusive. As a step toward enhancing risk management, this study serves as a baseline to grasp the current state of affairs, leveraging firsthand information. The methodology detailed herein can be cyclically employed to gage and oversee the effectiveness of risk mitigation and prevention strategies. The visual outcome of this study refers to the spatial depiction of vulnerability distribution across the area. This will empower decision-makers and policymakers to craft preventive strategies that are specific to the needs of the local populace.

The implications of this study extend widely to both policy formulation and future research pursuits. The evaluation of vulnerability to debris flows in the Pampa del Carmen sector illuminates the urgency for robust risk management strategies and accentuates the pivotal significance of intercultural perspectives in such evaluations. In future, policymakers can assimilate the insights reported in this study into their disaster risk reduction blueprints to ensure that intercultural dimensions are intricately woven into policies directed at improving community resilience. Moreover, forthcoming research endeavors could delve more profoundly into the intricacies of intercultural vulnerability dynamics, probing into targeted methodologies to address specific challenges encountered by heterogeneous communities. This study establishes a solid foundation for an all-encompassing approach to disaster risk reduction, and its findings act as a driving force for further inquiries that bear the potential to positively impact the well-being and safety of vulnerable populations.

Despite the valuable insights into the vulnerability assessment of the Pampa del Carmen sector, this study poses certain limitations. First, the assessment primarily relied on self-reported data from residents, which could introduce response bias and inaccuracies. Although we attempted to ensure accurate information, the subjectivity in reporting cannot be completely eliminated. Second, the assessment focused on a specific geographical area, and the findings may not be directly applicable to other regions with vastly varying sociocultural and environmental characteristics. Finally, the intercultural dimensions considered in this study, regardless of being critical, may be influenced by complexities that are difficult to capture comprehensively in a single assessment. Thus, future research should address these limitations by employing diverse data sources, extending the geographical scope, and further delving into the intricate interplay between intercultural factors and vulnerability dynamics.

## Data availability

Data available on request from the authors.

## CRediT authorship contribution statement

**Luis Izquierdo-Horna:** Conceptualization, Formal analysis, Funding acquisition, Investigation, Methodology, Project administration, Resources, Supervision, Writing – original draft, Writing – review & editing, Validation, Visualization. **Angelica Sánchez-Castro:** Conceptualization, Formal analysis, Investigation, Methodology, Writing – original draft, Data curation, Validation, Resources, Software. **Jose Duran:** Formal analysis, Investigation, Methodology, Software, Validation, Data curation, Resources, Writing – original draft.

## Declaration of competing interest

The authors declare that they have no known competing financial interests or personal relationships that could have appeared to influence the work reported in this paper.
